# The interactions of folate with the enzyme furin: a computational study

**DOI:** 10.1039/d1ra03299b

**Published:** 2021-07-06

**Authors:** Zahra Sheybani, Maryam Heydari Dokoohaki, Manica Negahdaripour, Mehdi Dehdashti, Hassan Zolghadr, Mohsen Moghadami, Seyed Masoom Masoompour, Amin Reza Zolghadr

**Affiliations:** Department of Internal Medicine, Aliasghar Hospital, Shiraz University of Medical Sciences Shiraz Iran; Department of Chemistry, Shiraz University Shiraz 71946-84795 Iran arzolghadr@shirazu.ac.ir +98 713 646 0788 +98 713 613 7100; Pharmaceutical Sciences Research Center, Shiraz University of Medical Sciences Shiraz Iran; Department of Pharmaceutical Biotechnology, School of Pharmacy, Shiraz University of Medical Sciences Shiraz Iran; Microbiology Laboratory, Moslemin Hospital Shiraz Iran; Medical School, Shiraz University of Medical Sciences Shiraz Iran; Non-Communicable Diseases Research Center, Shiraz University of Medical Sciences Shiraz Iran; Fars Science and Technology Park Shiraz Iran

## Abstract

Entrance of coronavirus into cells happens through the spike proteins on the virus surface, for which the spike protein should be cleaved into S1 and S2 domains. This cleavage is mediated by furin, a member of the proprotein convertases family, which can specifically cleave Arg-X-X-Arg↓ sites of the substrates. Here, folate (folic acid), a water-soluble B vitamin, is introduced for the inhibition of furin activity. Therefore, molecular insight into the prevention of furin activity in the presence of folic acid derivatives is presented. To this aim, molecular docking, molecular dynamics (MD) simulations, and binding free energy calculations were performed to clarify the inhibitory mechanism of these compounds. In this regard, molecular docking studies were conducted to probe the furin binding sites of folic acid derivatives. The MD simulation results indicated that these drugs can efficiently bind to the furin active site. While the folic acid molecule tended to be positioned slightly towards the Glu271, Tyr313, Ala532, Gln488, and Asp530 amino acids of furin at short and long ranges, the folinic acid molecule interacted with Glu271, Ser311, Arg490, Gln488, and Lys499 amino acids. Consequently, binding free energy calculations illustrated that folic acid (−27.90 kcal mol^−1^) has better binding in comparison with folinic acid (−12.84 kcal mol^−1^).

## Abstract 2

The present study introduces the ability of folic acid to interact and inhibit furin proprotein.

## Graphical abstract

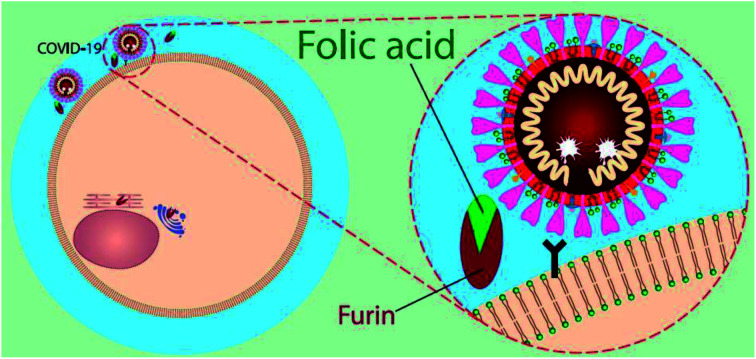

## Introduction

Coronaviruses, a family of Coronaviridae, can cause significant human pathologies such as respiratory tract infections in humans and other mammals.^1^ Coronavirus infections are usually mild, but some beta coronaviruses including Middle East respiratory syndrome coronavirus (MERS-CoV) and severe acute respiratory syndrome coronavirus (SARS-CoV) may induce critical symptoms.^2,3^

In December 2019, an outbreak of lower respiratory tract infections was reported in Wuhan, China.^4^ The pathogen was recognized as a novel RNA beta coronavirus, later named as SARS-CoV-2.^5^ The infection caused by this virus, COVID-19, has been declared by the World Health Organization (WHO) as a pandemic.^6^ In view of the novelty of SARS-CoV-2, further studies are required to obtain more insights about its pathogenesis.

The coronavirus (CoV) genome encodes four structural proteins, comprising spike (S), membrane (M), envelope (E), and nucleocapsid (N). The spike (S) protein of coronaviruses mediates receptor binding and fusion of the virus with the target cells.^7^ Each class of coronavirus attaches to a specific cellular receptor to facilitate virus entrance into cells. The angiotensin-converting enzyme 2 (ACE2) and CD209L are shown responsible for SARS-CoV entrance.^8,9^ It is reported that SARS-CoV-2 enters the respiratory tract by interacting with the ACE2 receptor.^10^

**Fig. 1:**
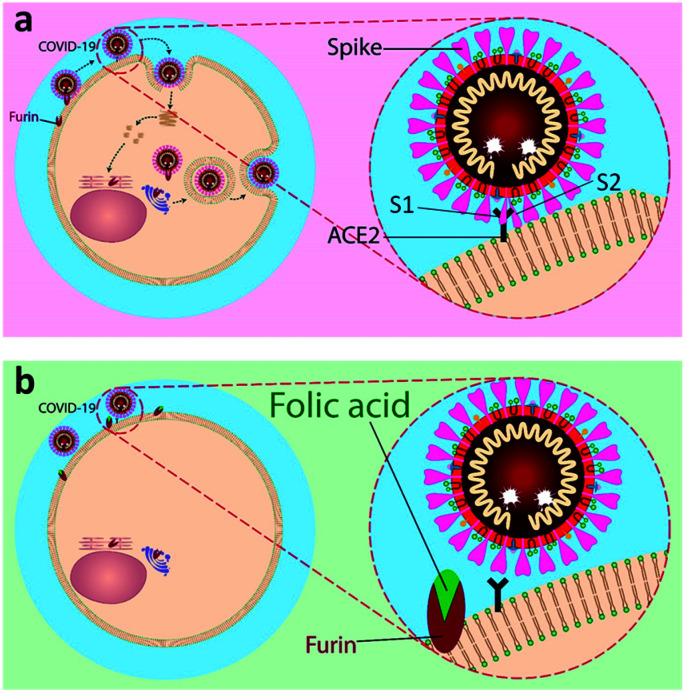
A schematic representation of inhibitory action of folic acid. (a) The mechanism of fusion and replication of COVID-19 virus. (b) Inhibition of furin protein by folic acid.

The spike protein comprises an amino (N)-terminal S1 subunit and a carboxyl (C)-terminal S2 subunit. The entrance of the virus is facilitated by cleavage of S protein to S1/S2 subunits. The S1 subunit binds to the ACE2 receptor, while the S2 site interacts with the cell membrane to mediate receptor-dependent endocytosis,^11^ as shown in Fig. 1a. The coronavirus spike protein is cleaved into S1 (receptor binding subunit) and S2 (membrane fusion subunit) by a proteolytic activation at the furin consensus motif RRRR_537_↓S (R = arginine, ↓: cleavage site) in virus-infected cells. Additionally, the S2 subunit of the S protein is further cleaved at the second furin site (RRRR_690_↓S) in the infected cells expressing S constructs.^12–16^ Mutations of one basic residue in the RRRR_690_↓S motif and use of non-furin cleavable PRRR↓S sequence demonstrated that furin may play an important role in furin-dependent entry.^17^ The working protease is a cellular proprotein convertase that circulates between plasma membrane, early endosome, and trans-Golgi network (TGN), by participation in endocytic and exocytic paths.^18,19^ This proprotein convertase is a major candidate for processing the surface glycoproteins of pathogenic viruses.^20,21^ Furin can cleave precursor proteins with specific motifs to produce mature proteins with biological activity. The first (P1) and fourth (P4) amino acids at the N-terminus of the substrate cleavage site must be arginine “Arg-X-X-Arg ↓” (R-X-X-R, X = any amino acid, ↓: cleavage site). If the P2 position is basic lysine or arginine, the cleavage efficiency could be improved by about 10 times.^22^ The results of a series of analyses have proposed that one of the important reasons for the high infectivity of COVID-19 is a redundant furin cut site in the virus spike protein.^23^

Our aim is to suggest folic acid as a potential inexpensive, safe, and non-immunogenic drug candidate for the prevention or treatment of early stages of respiratory disease associated with COVID-19 (Fig. 1). Folic acid is a type of B vitamin normally found in foods such as spinach, broccoli, asparagus, dried beans, lentils, peas, and oranges. Folic acid helps the body produce and maintain new cells and also prevent changes to DNA that may lead to cancer. Noticeably, folic acid deficiency is associated with a variety of human malignancies, including colorectal cancer. The over-expression of folate receptors in the early stages of malignant cell formation can be due to folic acid deficiency. Besides, folate malnutrition can cause a high incidence of adenomatous polyps and premalignant lesions of the colon.^24^ To this aim, the molecular dynamics (MD) simulations of the interactions of furin enzyme with folic acid and one of its active metabolites, folinic acid, was performed here for the first time to evaluate the interplay of these molecules with furin.

## Computational methods

### Molecular dockings

**Fig. 2:**
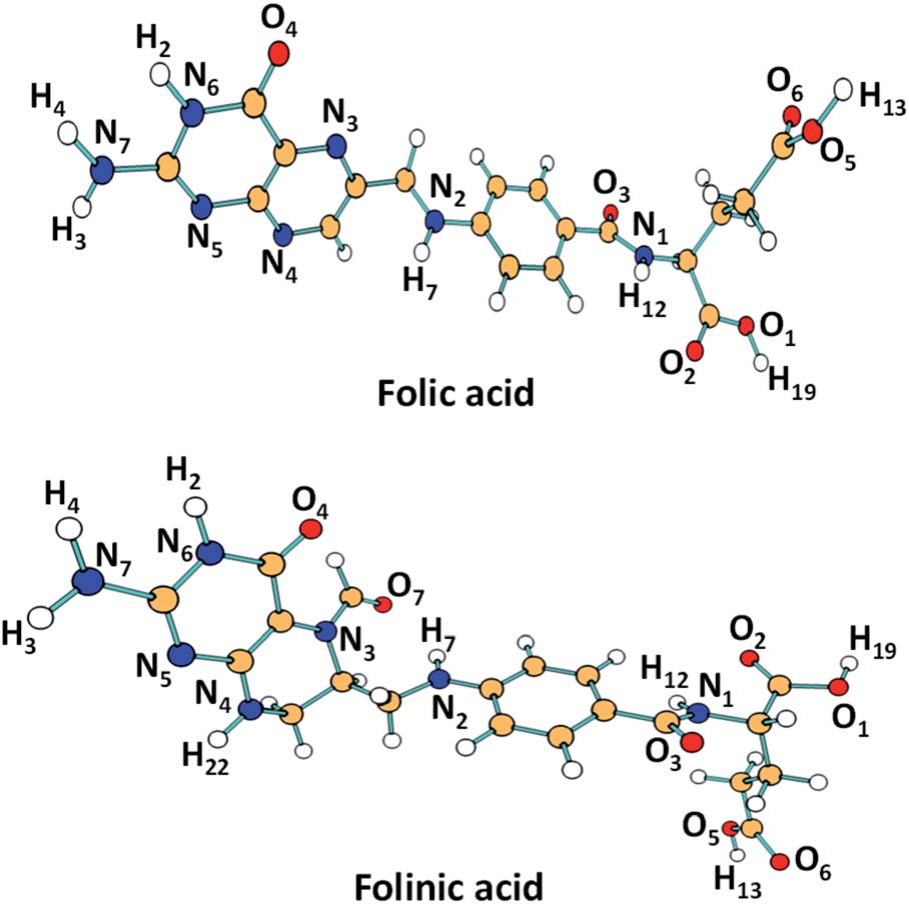
DFT-calculated geometry-optimized (B3LYP/6-31+G(d,p)) structures of drug molecules with atoms labeling discussed in the molecular analysis.

In this study, docking of two folate analogs, folic acid and folinic acid against human furin was performed using molegro virtual docker (MVD) version 6.0 software.^25^ An X-ray crystal structure of human furin was used for docking studies taken from the Protein Data Bank (PDB code 5MIM) accessed at the URL (http://www.rscb.org/pdb) with reasonable resolution (≤1.9 Å). The optimized coordinates (see Fig. 2) of folic acid and folinic acid were obtained by DFT calculations at the B3LYP/6-311+G** level of theory implemented in Gaussian software.^26^ Two drugs were docked against furin protein and 100 independent runs were performed with the guided differential evolution algorithm. In case of furin–drug complexes, the program generally identified five different binding sites. Among these five predicted cavities, the one with the volume of 75.776 Å^3^ was selected as the potential binding site for investigation. For each docking of protein–ligand, 10 docked poses were generated and listed in Table 1. The Moldock score of the best drug–furin complex of folic acid, and folinic acid were −140.40, and −136.90 kcal mol^−1^, respectively.

**Table 1:** MolDock score, Re-rank score and the hydrogen bond (H-bond) and interaction energies (kcal mol^−1^) of the docked compounds

Ligand	Docked pose	MolDock score	Re-rank score	H-Bond energy	Interaction energy
Folic acid	1	−138.81	−115.96	−8.41	−147.46
	2	−140.40	−122.44	−14.27	−161.60
	3	−138.66	−113.85	−7.9	−157.31
	4	−139.41	−105.99	−8.24	−149.05
	5	−138.22	−117.37	−8.55	−155.33
	6	−133.07	−98.85	−10.17	−143.51
	7	−125.00	−96.49	−11.99	−153.20
	8	−124.16	−99.49	−12.22	−143.89
	9	−127.54	−106.55	−5.72	−142.12
	10	−132.35	−95.14	−5.71	−156.08
Folinic acid	1	−136.15	−105.49	−12.79	−154.25
	2	−131.43	−80.03	−8.74	−138.25
	3	−136.90	−115.08	−13.85	−159.20
	4	−131.00	−104.63	−11.56	−145.97
	5	−121.98	−109.21	−9.80	−147.72
	6	−120.31	−90.47	−11.01	−128.46
	7	−129.95	−103.88	−6.67	−145.28
	8	−123.30	−75.43	−3.67	−139.83
	9	−115.99	−76.49	−3.30	−137.69
	10	−118.37	−93.77	−6.26	−137.29

### Molecular dynamics simulations in water

The best configurations from the above-mentioned procedures were selected as the initial structures for MD simulation in aqueous media. In this case, the MD simulations were performed with the GROMACS 4.5.4 program using the GROMOS96 53A6 force field employing periodic boundary conditions in three dimensions.^27^ The simulations consisted of three different systems: (I) furin in water (furin) as control system, (II) furin–folic acid in water (furin/folic acid), and (III) furin–folinic acid in water (furin/folinic acid). The partial atomic charges were calculated by using natural population analysis as implemented in Gaussian 09 program. All simulations were performed in the presence of water by using simple point charge (SPC) model.^28^ The net charge of the systems was neutralized by addition of the same amount of sodium or calcium ions. After energy minimization, an equilibration *NVT* run has been carried out over 500 ps, while restraining the position of furin by force constant of 1000 kJ mol^−1^ nm^−2^ to their initial position. The systems were simulated for 1 ns using an *NPT* ensemble. Finally, production runs have been conducted over 500 ns with time steps of 2 × 10^−3^ ps at 310 K. The first 100 ns of trajectories were set aside for equilibration and were discarded during analysis.

### Binding free energy calculations using MM-PBSA

The binding free energies between drug molecules and furin were calculated by using Molecular Mechanics Poisson–Boltzmann Surface Area (MM-PBSA) method to comparatively evaluate binding ability of inhibitors to furin using the g_mmpbsa tool of GROMACS.^29^ In this regard, binding free energy was evaluated based on the following equations:Δ*G*_binding_ = *G*_complex_ − (*G*_protein_ + *G*_ligand_) =Δ*E*_MM_ + Δ*G*_solv_in which *G*_complex_, *G*_protein_ and *G*_ligand_ indicate free energies of the docked complex, protein, and drug, respectively. Molecular mechanical (MM) energy, Δ*E*_MM_ represents the sum of electrostatic and van der Waals interactions of inhibitors with proteins, independently (Δ*E*_MM_ = Δ*E*_elec_ + Δ*E*_vdW_). The solvation free energy, Δ*G*_solv_ was identified based on the equation of Δ*G*_solv_ = Δ*G*_pol_ + Δ*G*_nonpol_, in which Δ*G*_pol_ and Δ*G*_nonpol_ are the polar and nonpolar solvation free energies, respectively. In agreement with a number of previous computational investigations,^30–32^ the contribution of conformational entropy of molecules was ignored in the reported relative Δ*G*_binding_.

## Results

### Evaluation of the binding modes using molecular docking

**Fig. 3:**
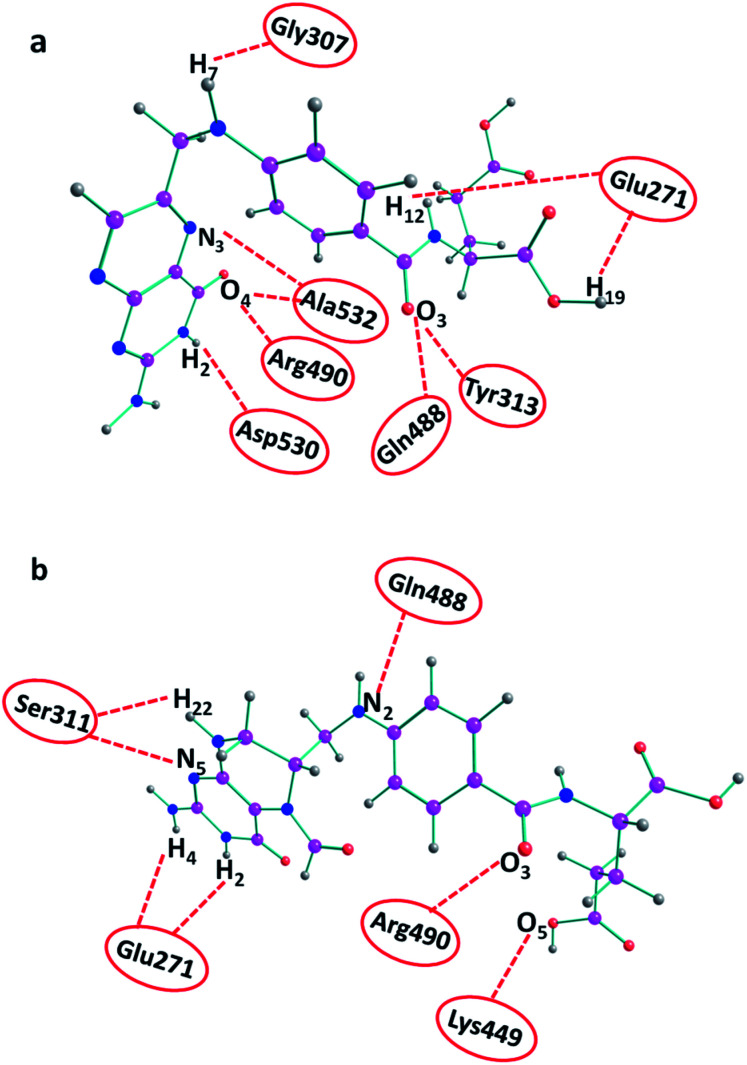
H-bond interactions of drugs with various amino acids of furin obtained by docking. (a) Folic acid and (b) folinic acid.

The ligand–furin docking was performed to predict the major binding sites of folic acid and folinic acid molecules in the active site of furin protein. In general, the interaction energies between protein and ligands are obtained by docking studies. The results showed that folic acid and folinic acid molecules interacted well with the active site residues of furin by formation of hydrogen bonds (H-bond). Different atom sites of the two drug molecules established hydrogen bonding interactions with various amino acids of furin as shown in Fig. 3. Interestingly, the binding sites of folinic acid and furin were clearly different. The interactions of Gly307, Glu271, Tyr313, Gln488, Ala532, Arg490, and Asp530 residues with hydrogen, oxygen, and nitrogen atoms of folic acid were dominant; whereas, strong H-bonds were established through Ser311, Glu271, Arg490, Lys449, and Gln488 residues of folinic acid molecules. Moreover, while the H_19_ and H_12_ atoms of folic acid interacted substantially with Glu271, the H_2_ and H_4_ atoms of folinic acid formed H-bonds with the Glu271 residue of furin. These findings proposed different orientation preferences of folic acid and folinic acid molecules in the binding site of furin. As observed in Table 1, the folic acid and folinic acid drugs showed the highest H-bond energy of −14.27 kcal mol^−1^ and −13.85 kcal mol^−1^, respectively. Furthermore, the obtained interaction energies were −161.60, and −159.20 kcal mol^−1^ for folic acid and folinic acid, respectively.

### Elucidation of inhibitors/furin interactions by MD simulations

**Fig. 4:**
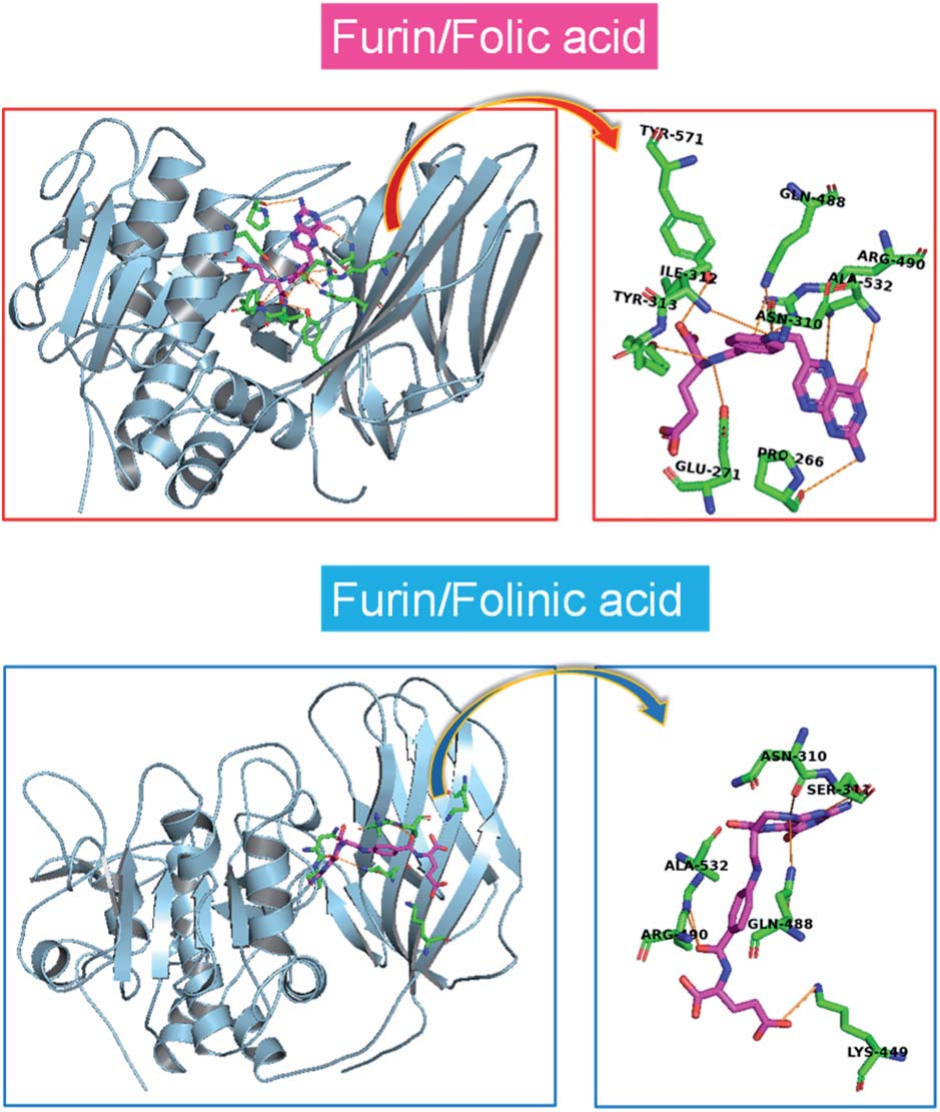
One mode of drugs binding to the furin protein taken from a snapshot of the simulation at 400 ns. Water molecules were removed for clarity.

The MD simulations of folate derivatives interaction with furin were also conducted to gain additional insight into the specific mechanism by which folic acid and folinic acid molecules can exert their potential inhibitory actions in the furin active sites of COVID-19 patients. Atoms labeling for the two drug molecules under study are shown in Fig. 2. The snapshots of the simulated systems after 400 ns (water molecules were removed for clarity) are depicted in Fig. 4. The position and orientation of drug molecules in the simulations are in line with docking results. The intermolecular interactions between different residues of furin and drug molecules were studied here.

**Fig. 5:**
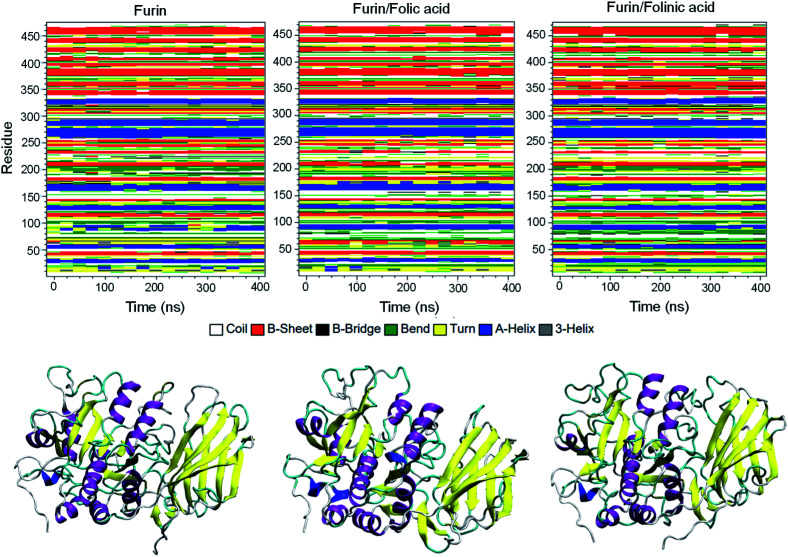
Secondary structures of furin for each system. Secondary structure of unbound furin and furin bound to drugs displayed in ribbon representation.

**Fig. 6:**
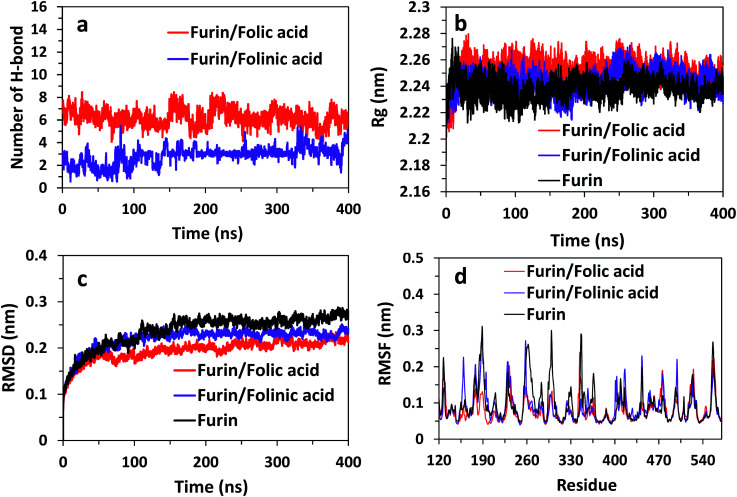
(a) Number of H-bonds for systems over the last 400 ns of simulations. *R*_g_, RMSD, and RMSF of furin structure in three systems are shown in panel (b)–(d).

To obtain the influence of drug molecules on the overall stability of furin structure, a comparative structural evaluation of furin as control system and furin–folic acid and furin/folinic acid as complex systems has been performed. Throughout the MD simulations, both drugs affect the furin secondary structures (see Fig. 5 and Table 2). Furin in all systems is largely dominated by the coil, β-sheet and α-helix structures. In this regard, the average of number of H-bonds between folic acid and furin were obtained to be 6.25 ± 1.20 per time frame, relative to the value of 2.86 ± 0.96 H-bonds per time frame for folinic acid (see Fig. 6a).

**Table 2:** The secondary structural component statistics of furin and furin complexes during MD simulation in water

Secondary structure component	Furin	Furin/folic acid	Furin/folinic acid
Coil	30	31	30
β-Sheet	25	28	26
β-Bridge	2	1	2
Bend	16	10	15
Turn	11	13	11
Helix^a^	16	17	16

a Helix is the sum of α-helix, π-helix and 3_10_-helix.

The changes in the structural stability of furin is measured for each system by root-mean-square deviations (RMSD), root-mean-square fluctuations (RMSF), to observe fluctuations in amino acid residues, and radius of gyration (*R*_g_). The *R*_g_ was calculated to examine the compactness of protein structure in the presence of inhibitors.

The results are shown in Fig. 6b indicate that *R*_g_ value is slightly higher for complex systems as compared with control system. For instance, the *R*_g_ fluctuates to the value ∼2.26 nm for furin/folic acid system, and ∼2.24 nm for furin system. Thus, ligands induces furin structure to adopt less compact conformations in water in contrast to control system. RMSD of furin in all three systems is shown in Fig. 6c. We see that RMSD of control system converges around ∼0.26 nm. RMSD fluctuates to a lower value for furin–folic acid complex (∼0.21 nm) as compared with furin–folinic acid complex (∼0.23 nm). In order to evaluate structural flexibility of furin, RMSFs of residues are calculated and shown in Fig. 6d. For better clarity, RMSFs of C_α_ atoms of residues in furin were computed for all systems. Generally, the RMSFs of the whole regions are decreased relative to the furin in control system, which shows that the structural flexibility of residues are diminished by binding of inhibitors. Thus, the furin–drug complexes are likely to remain more stable than that of free furin.

### Distribution functions

**Fig. 7:**
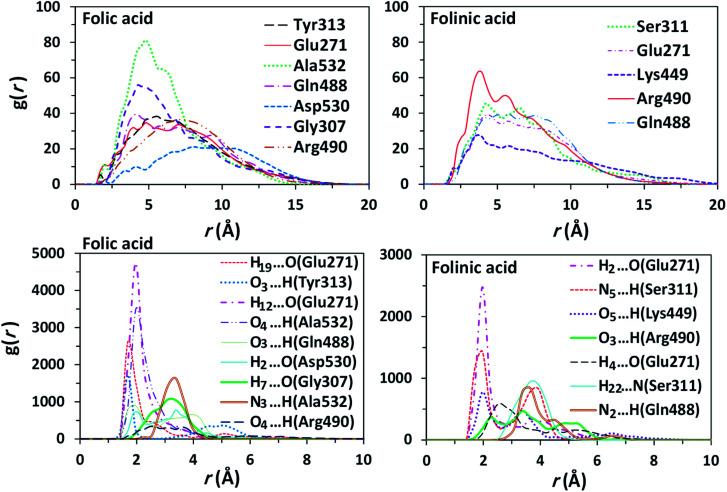
Comparison of RDFs between the centers of mass of some residues of furin with drugs (top panel) and the atom…atom RDFs (bottom panel) for systems contain: folic acid (left panel), folinic acid (right panel).

**Fig. 8:**
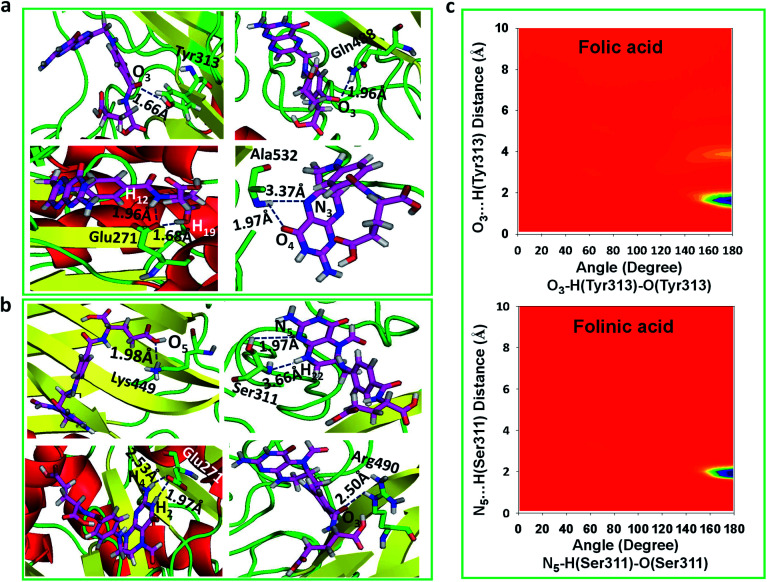
The main interaction of drugs with different residues of furin. (a) Folic acid. (b) Folinic acid. Drug molecules and residues are represented with sticks. For drugs, the red, blue, pink and white atom sites represent the oxygen, nitrogen, carbon, and hydrogen atoms. For amino acids of furin, the carbon atoms are colored in green. (c) CDF of (up panel) furin–folic acid, (down panel) furin–folinic acid systems.

To obtain statistically reliable structural data, the radial distribution functions (RDFs) are calculated by averaging over trajectories of long production runs. The RDFs between furin's residues and drug molecules are shown in Fig. 7. For clarity, visual inspection of interaction sites are also demonstrated in Fig. 8 (see Fig. 2 for atom's labels). The RDFs between the center of mass of folic acid and different amino acids of furin indicates that Ala532, Tyr313, and Glu271 interacted with folic acid with a relatively high probability and small dynamics both at short and long distances. In this case, the atom…atom RDFs demonstrated that position of the first peak obtained for H_19_⋯O(Glu271) and O_3_⋯H(Tyr313) were smaller than that of O_4_⋯H(Ala532). Interestingly, MD simulations showed that the distance of O_3_ atom of folic acid with Tyr313 and Gln488 hydrogen atoms were calculated to be 1.66 Å, and 1.96 Å, respectively (see Fig. 2 for atom labeling and Fig. 8a). The main interaction of O_4_ atom of folic acid with Ala532, was located at 1.97 Å. The RDF peaks in Fig. 7 (right panel) showed that the folinic acid tended positioning slightly towards Ser311, Glu271, Arg490, Gln488, and Lys499 at short and long ranges. As shown in Fig. 8b, the main atomic interactions in this case were H_2_⋯O(Glu271), and N_5_⋯H(Ser311), which were located at 1.97 Å. More details can be obtained from the spatial distribution function of the hydrogen bonding between the furin's amino acids and different atom sites of the drug molecules by the calculation of combined radial/angular distribution function (CDF) as a powerful tool for defining H-bond criteria.^33^Fig. 8c (up panel) manifests the most favorable hydrogen bonding interaction between the polar hydrogen atoms of the Tyr313 and the O_3_ atom of carbonyl group of folic acid in the angle range 170 < *θ* < 180 at the distance of around 1.66 Å. Particularly, the CDF of Fig. 7c (down panel) indicates that there are interactions between the hydroxyl group of Ser311 and the folinic acid with the angle range 170 < *θ* < 180 and 1.97 Å distance. These interactions occurred when the drug molecules tilted substantially to directly interact with furin. Based on these findings, the folic acid…furin interactions were more probable than folinic acid.

### Binding free energies

To get an insight into molecular interactions between inhibitors and furin protein in aqueous media, binding free energy for each protein–ligand complex was evaluated using MM-PBSA method. As shown in Table 3, the binding free energy calculations indicate that the binding is an energetically favorable process for both drug molecules. Binding free energies clearly depicted that folic acid had higher binding energy (Δ*G*_binding_ = −27.90 kcal mol^−1^) as compared to folinic acid (Δ*G*_binding_ = −12.84 kcal mol^−1^). From the data reported in Table 3, non-bonded van der Waals, non-bonded electrostatic interactions, and non-polar component to solvation are noted to be favorable for both complexes. Δ*E*_elec_ values are stronger than Δ*E*_vdW_ values for both systems. This result highlight that electrostatic interactions between folic acid (folinic acid) and furin dominate over the van der Waals interactions. Thus, intermolecular nonpolar solvation and electrostatic interactions are the main forces involved in the binding of drugs with furin structure. The binding affinity (dissociation constant (*K*_d_)) of folate for human folate receptor *via* isothermal titration calorimetry measurements has been obtained to be ∼10 × 10^−12^ M.^34^ Also, the binding affinity of folate receptor toward folate measured by biolayer interferometry was ∼1.14 × 10^−9^ M.^35^ Folic acid binding to folate receptor determined by saturation radioligand-binding assay has been examined to be ∼1.90 × 10^−10^ M.^36^ The binding free energy of protein-ligand complex formation, can be associated with the dissociation constant through 
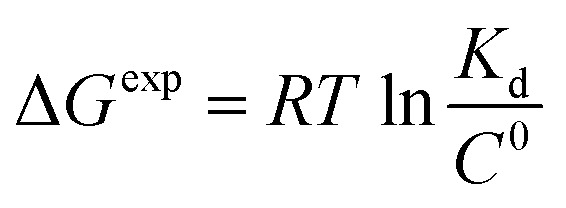
 equation, in which *T* is the absolute temperature, *R* is the ideal gas constant, *C*^0^ is generally set to 1 with the moles per liter dimension and 
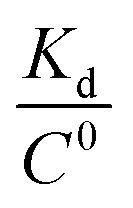
 is accordingly dimensionless. By using this equation, binding free energies of folate for human folate receptor were determined to be −15.05, −12.23, and −13.30 kcal mol^−1^, experimentally. Herein, the binding free energy of furin–folate complex predicted by MM-PBSA method is higher than the experimentally determined values of folate receptor–folate complex. It is encouraging that the rank of our predicted binding free energies is in agreement with the one determined by the experimental data.

**Table 3:** The binding free energy (kcal mol^−1^) of complex systems

Energy terms (kcal mol^−1^)	Folic acid	Folinic acid
Δ*E*_vdW_	−33.29 ± 3.14	−34.09 ± 2.63
Δ*E*_elec_	−49.64 ± 5.96	−63.53 ± 4.31
Δ*E*_MM_^a^	−82.93 ± 5.85	−97.62 ± 5.22
Δ*G*_pol_	88.99 ± 7.72	144.07 ± 3.50
Δ*G*_nonpol_	−32.96 ± 5.03	−33.61 ± 4.62
Δ*G*_solv_^b^	56.03 ± 6.75	110.46 ± 4.81
Δ*G*_binding_^c^	−27.90 ± 3.64	−12.84 ± 2.54

a Δ*E*_MM_ = Δ*E*_vdW_ + Δ*E*_elec_.

b Δ*G*_solv_ = Δ*G*_pol_ + Δ*G*_nonpol_.

c Δ*G*_binding_ = Δ*E*_MM_ + Δ*G*_solv_.

## Discussion

Furin enzyme is associated with a great number of pathologies, including bacterial and viral infections, cancer, and metastasis. Hence, this protein is extremely considered as a drug target.^37,38^ A characteristic feature of furin in the protease family is its very limited reactivity toward typical covalent inhibitors due to spatial restrictions.^39^ Previous studies have documented that furin could promote the activation of coronavirus by sequence-specific cleavage of the spike protein. Furin cleaves a wide variety of protein precursors in the preferred consensus motif RXR(K)R/R (R = arginine, K = lysine, X= any amino acid).^12,40^ Therefore, furin protein appears to be a promising target for the infection treatment. The present study identified folic acid as a furin-binding protein by using an all atom MD simulation study. It is well known that folate receptors are mainly expressed in lungs and kidney in normal conditions.^41^ Interestingly, the ACE2 receptors are also mostly expressed in lung. Recent studies have proposed that furin inhibition can have a substantial role in the prevention of COVID-19 infection progress.^42^ Folic acid is small and stable over a broad range of temperatures and pH values, and it retains its ability to bind to the folate receptor after conjugation with drugs or diagnostic markers.^43,44^ The present study introduces the ability of folic acid to interact and inhibit furin proprotein.

In this study, structural parameters such as radial distribution functions and combined radial-angular distribution functions were used to analyze the intermolecular interactions between folic acid (or folinic acid) and furin protein. The RDF presents the probability of finding a particle at a certain distance from another reference particle, therefore, contains the information of the average nearest neighbors' distance.^45,46^ The first peaks of simulated RDFs were positioned at very short distances with very high probabilities, which could be attributed to the strong intermolecular interaction between folic acid molecule and furin enzyme. The combined distribution functions confirmed the H-bond character of folic acid–furin interactions. The results indicated that the interactions between folinic acid and furin were high, but substantially lower than that of folic acid. Binding affinities of inhibitors to furin were computed and the results show that binding ability of folic acid is stronger than folinic acid. In this way, folic acid could block the access of COVID-19 spikes to furin and prevent the cell entry and consequently turn-over of the virus.

In summary, our results suggest that folic acid could be used to inhibit the furin enzyme. The association of folic acid with furin would affect the structure of the protein and consequently interfere with its proteolytic capability. Thus, folic acid, as a safe drug, could be useful in the prevention or management of COVID-19-associated respiratory disease in the early stages of the disease.

## Conclusions

In the present study, the effect of folate derivatives as safe active inhibitors on the structural stability of furin protein was investigated using molecular docking and equilibrated trajectories of MD simulations. The results indicate that binding of folate derivatives leads to the conformational changes of the protein and also affects its internal dynamics. The radial and combined distribution functions reveal that the main interaction between furin and drug molecules is through hydrogen bonding formation. The binding free energy analysis between inhibitors and furin structure with MM-PBSA method inferred that folic acid has better binding affinity as compared to folinic acid. This insights into the underlying inhibitory mechanism of folic acid that show potential inhibitory activity against furin will be beneficial for the current and future COVID-19-associated respiratory disease therapeutic studies.

## Conflicts of interest

There are no conflicts to declare.

## Supplementary Material
